# Physical Workload Tracking Using Human Activity Recognition with Wearable Devices

**DOI:** 10.3390/s20010039

**Published:** 2019-12-19

**Authors:** Jose Manjarres, Pedro Narvaez, Kelly Gasser, Winston Percybrooks, Mauricio Pardo

**Affiliations:** Department of Electrical and Electronics Engineering, Universidad del Norte, Barranquilla 081001, Colombia; pjnarvaez@uninorte.edu.co (P.N.); kgasser@uninorte.edu.co (K.G.); wpercyb@uninorte.edu.co (W.P.); mpardo@uninorte.edu.co (M.P.)

**Keywords:** human activity recognition, physical workload, wearable systems for healthcare, machine learning for real-time applications

## Abstract

In this work, authors address workload computation combining human activity recognition and heart rate measurements to establish a scalable framework for health at work and fitness-related applications. The proposed architecture consists of two wearable sensors: one for motion, and another for heart rate. The system employs machine learning algorithms to determine the activity performed by a user, and takes a concept from ergonomics, the Frimat’s score, to compute the corresponding physical workload from measured heart rate values providing in addition a qualitative description of the workload. A random forest activity classifier is trained and validated with data from nine subjects, achieving an accuracy of 97.5%. Then, tests with 20 subjects show the reliability of the activity classifier, which keeps an accuracy up to 92% during real-time testing. Additionally, a single-subject twenty-day physical workload tracking case study evinces the system capabilities to detect body adaptation to a custom exercise routine. The proposed system enables remote and multi-user workload monitoring, which facilitates the job for experts in ergonomics and workplace health.

## 1. Introduction

According to the World Health Organization (WHO), the amount of workload can be a hazard at the workplace leading to work-related stress [1]. Having too much or too little to do at work is often an indication of bad time management that results on mental stress [1,2]. Mental stress affects the heart rate (HR) that in turns spreads its effects to other parts of the body [3]. Workload is a key factor in ergonomics to determine the adequate length and number of rest breaks for a given job, helping to reduce work-related stress [4]. However, the amount of physical workload is not necessarily determined by the length of a particular task, but by the quantity of energy required to complete it, and can also be reflected on the HR [5].

In consequence, works like [5] describe the importance of HR tracking in physical workload assessment. The authors of [5] perform a comparison between using absolute cardiac cost (ACC) and relative cardiac cost (RCC) to evaluate physical workload based on HR values during resting periods between activities. Similarly, Solé proposes in [6] an standardization for workload values based on RCC using the Chamoux [7] and Frimat [8] criteria, where the numeric workload scores are mapped into categories going from *extremely hard* to *very light*. These criteria allow a qualitative assessment of workload using only HR measurements.

A common element among the physical effort assessment systems is HR tracking. HR has a well-known relation with mental stress, as evidenced in [9,10,11,12]. The methods to obtain information about heart activity must be reliable and must allow their implementation using non-invasive devices to be relevant in practice. In [13], a comparison of HR signals coming from an electrocardiography (ECG) and a photoplethysmography (PPG) sensor establishes the reliability of PPG to obtain HR information. Additionally, the authors of [14] validate the use of a commercial HR monitor, which employs PPG to track HR waveforms during rest. A similar conclusion is found in [15] with a smartwatch. This validation of PPG-based HR tracking has led to developments that seek to strengthen HR monitoring on environments where sensor signals can be corrupted by body movements [16]. Despite of such possible corruption, other systems have been built with PPG sensors under movement conditions and have not displayed issues regarding performance [17,18,19].

Regarding physical workload, several methods for continuous track of the physical effort can be found in literature where qualitative data are not provided. For example, in [20] Jovanov et al. use a wireless body area network (WBAN) to monitor motion from on-body accelerometer and electrocardiography (ECG) sensors. Such a system intends to keep track of the physical activity and health status with non-invasive technology. Other works like [21,22,23,24] propose manually initiated recording of activities with data from HR trackers to measure the physical workload of a given population. Such a method is tested in [21,22,23,24] with salsa dancers, dockers, nurses and porters, respectively. Complementary, Jovanov et al. introduce real-time HR monitoring and step counting to track the work stress in nurses in [12]. Our previous work in [19] improves the method proposed in [21] by developing a mobile application that computes the workload during each activity performed by janitorial staff. In the case of [19] the system is manually initiated, as in [21,22,23,24], and allows only local monitoring, which requires the presence of an expert next to the worker. In most real-life scenarios, having an ergonomics expert all over the workplace is unfeasible.

To follow the tendencies on mobile-health applications, it is necessary to address the problem of workload assessment from a real-time perspective as suggested by [12]. Therefore, HR tracking must be integrated with real-time human activity recognition (HAR) or online HAR (according to [25]) to achieve workload assessment without requiring manual intervention to indicate the start and end of an activity. Some works have shown efforts to achieve this integration. For example, [26,27,28] exhibit systems that combine HR tracking and online HAR using on-body sensors and an integration device to receive and display the sensors information. The integration device, typically a smartphone, can take the place of a movement sensor [29]. Accordingly, [30,31,32] describe smartphone-based HAR systems along with their corresponding challenges regarding feature extraction and selection. However, these systems are highly dependent on the on-body location of the smartphone. On the other hand, accelerometer-based HAR architectures present robust performance regardless the sensor location and allow to distinguish among a wider range of activities compared to smartphone-based systems [26,33,34,35]. 

The robustness and reliability of HAR with accelerometers is reflected in the variety of the e-health applications where it is found. For example, [36] employs accelerometer-based HAR for posture recognition which helps to monitor falls in elderly people. Such an approach is also present in [37,38,39], which demonstrates the rising popularity of this application. Moreover, some developments related with sports and fitness complemented with HAR are shown in [40,41].

Thus from these previous works, it can be concluded that HAR is suitable as a key component for workload tracking. Nevertheless, the need of relevant implementations that integrate HR and HAR tracking and take it a step further to qualitative workload assessment remains a challenge from an ergonomic point-of-view. Consequently, the work described in this article presents a solution that embraces wearable technology and machine learning algorithms to compute physical workload in real-time. Our solution here combines the HAR and HR tracking to achieve a workload assessment that is linked automatically with the performed activity. Compared to our previous work in [19], this new system eliminates the need of an expert next to every single worker that is being tracked, since it enables remote and multi-user monitoring.

The rest of the paper is organized as follows. Section 2 describes the characteristics of the wearable devices implemented in this system, details the workflow of the mobile application, and displays how the system classifies the workload. Section 3 shows the development of the online HAR component and presents two cases of study to evaluate the online HAR performance and the physical workload assessment. Section 4 and Section 5 contain the discussion and conclusions regarding the results, respectively.

## 2. Materials and Methods

### 2.1. Wearable Devices and Mobile Application

**Figure 1:**
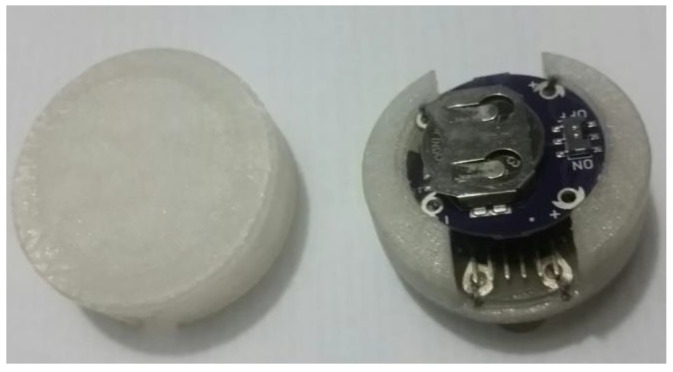
Human activity recognition hardware. The case allows the system to be worn on the hip.

The hardware for HAR comprises of an ultra-low power (ULP) microcontroller unit (MCU) with Bluetooth low energy (BLE) capability, an ULP MEMS (Micro Electro-Mechanical Systems) based accelerometer and a small Li-ion battery. The HAR hardware is displayed in Figure 1. The selected ULP MCU is the Lilypad Simblee, as used in [26]. The advantages of this device include small footprint (50 mm diameter), embedded BLE radio, and a battery charge controller. A 100 mAh Li-ion battery powers the Lilypad Simblee and can be recharged through a USB controller module [42]. This MCU samples the signal from a tri-axial accelerometer at a 20 Hz rate, as recommended in [25]. The accelerometer selection also follows the hardware used in [26], which is the ADXL335. This sensor allows us to obtain information from movement and inclination with a sampling frequency up to 50 Hz and an acceleration up to 2 *g* [43]. 

For the HR tracking, a Microsoft Band performs HR sampling with a built-in PPG sensor [44]. This wearable enables the tracking of other fitness-related variables such as sweating, arm movement and step counting, among others [44]. This device has been validated by different authors for HR monitoring [45,46]. For this work, we only required the HR sensor; and therefore, other sensors are deactivated to save energy. A specialized Software Development Kit (SDK) for Android devices permits the control of the Microsoft Band. This SDK can be found in [47]. We develop a mobile application that connects automatically to both sensors and has two operating modes: training mode and testing mode.

**Figure 2:**
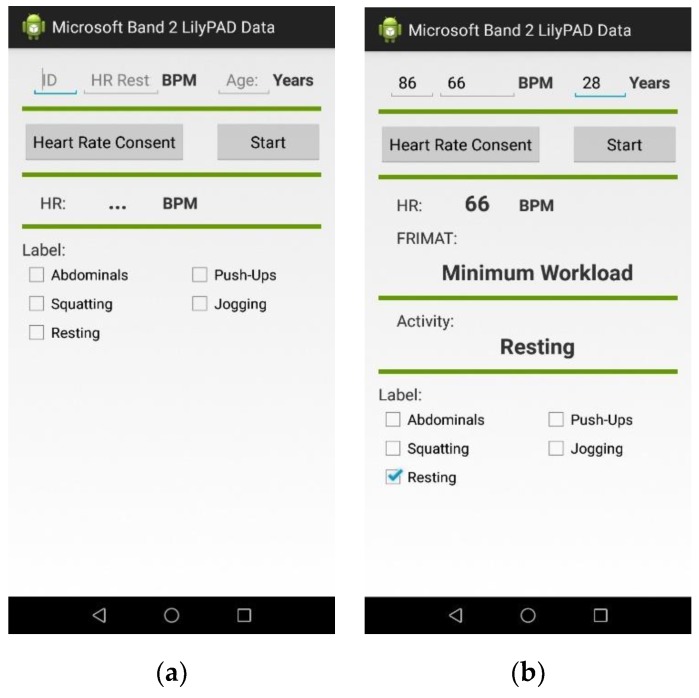
User interface of the mobile application. (**a**) Training mode. (**b**) Testing mode.

In training mode, the user interface (UI) asks for the activity that the user is going to perform from a list of predefined exercises (jogging, squatting, doing push-ups and doing crunches) and the average HR at rest to use it as a reference parameter for workload estimation. A 1-minute timer is used to standardize the length of the training sessions for the classification algorithms. The UI for this operation mode is displayed in Figure 2a. The application stores the incoming data from both sensors in a JSON (JavaScript Object Notation) array, expecting to have 20 samples of each accelerometer axis, the average HR within one second and a label representing the activity. Every second, the JSON array containing the sensor samples is sent to a cloud server for storage in a database. After taking training samples from nine subjects, a Python script retrieves the stored data along with its corresponding activity labels and trains a classification model using the scikit-learn library [48]. The details regarding training and validation are explained in the Section Results. Once the model is validated, the mobile application can function in the testing mode. In this mode, the sampling process from the sensors remains the same as in the training mode; however, the UI does not have any time restriction. Therefore, an array containing the samples from the tri-axial accelerometer is taken by a feature-computing function followed by the classification model and the average HR during activity passes through a workload estimator. Figure 2b shows the UI for the testing mode.

### 2.2. Physical Workload Computation

As [6] mentions, physical workload can be computed using metabolic consumption tables, oxygen consumption tables and HR measurements. However, HR measurements are the only non-invasive method, which allows the integration of wearable technology. In the literature, there are two criteria to evaluate HR-based workload: Frimat’s [8] and Chamoux’s [7]. In one hand, the Frimat’s criterion estimates workload on short work times or on specific activities; while, on the other hand, the Chamoux criterion computes the workload of a full workday (at least 8 h) [19]. For this work, the Frimat’s criterion is chosen since the target are fitness-related activities. The methodology used for physical workload computation is the same as in [19].

The selected method requires the computation of some cardiac indicators. The first one is the absolute cardiac cost (ACC) as defined by Equation (1),

(1) $$ACC=HR_{activity}-HR_{rest},$$

where *HR_activity_* refers to the average heart rate during the activity and *HR_rest_* is the statistical mode of the HR values measured during resting periods. ACC allows the estimation of intensity for a given task. Another indicator, the relative cardiac cost (RCC) is derived from the ACC as shown in Equation (2).

(2) $$RCC=\frac{ACC}{HR_{max}-HR_{rest}}\times 100.$$

RCC indicates the adaptation of the body to an activity.

In Equation (2), *HR_max_* stands for the maximum achievable HR by a subject. The exact value of *HR_max_* should be found in a stress test. However, [6] provides a theoretical definition, which can have up to 5% of error compared to the actual value. Such definition for *HR_max_* depends on the subject age as stated in Equation (3),

(3) $$HR_{max}=220-Age.$$

Frimat’s criterion also needs the calculation of the cardiac acceleration (ΔHR) defined in Equation (4), and the mean heart rate ($\bar{HR}$) within an arbitrary time window.

(4) $$\Delta HR=HR_{max}-\bar{\mathrm{HR}}.$$

Thus, once the five variables for Frimat’s criterion (ACC, RCC, *HR_max_*, $\bar{HR}$ and ΔHR) are computed, each one of them is mapped into a corresponding Frimat’s coefficient, which takes an integer value between 1 and 5. Table 1 details the relation between the values of each indicator and their respective Frimat’s coefficient.

Then, the method requires to take the Frimat’s coefficient from each input variable and add them up to obtain a Frimat’s score, which ranges between 5 and 25. This score is the value that determines the level of physical workload of an activity. Following the ranking presented in [6], an activity can be ranked as shown in Table 2. During the implementation of the workload computation, the system takes the resting HR that must be previously measured and compares it with the average HR within one-second time windows to compute the five cardiac indicators needed to obtain the Frimat’s Score. This score was mapped to its corresponding category according to Table 2, accompanied with the label of the most recent activity. 

## 3. Results

### 3.1. Training and Validation of the Activity Classifier

The implementation of the online HAR subsystem requires three critical steps: data collection, training and validation. To perform a reliable data collection, the selected activities must be clearly distinguishable from the sensor point-of-view and they should be related to a common set of tasks. For the sake of test subject availability, we selected a fitness routine, which includes jogging, doing crunches, push-ups and squatting. These activities are among the most common exercises performed by the local population. Since the workload assessment requires us to track the resting periods, standing still is also an activity into consideration. Additionally, to increase the system generalization capabilities, data collection must be done from heterogeneous sources, i.e., subjects with different anatomic characteristics and different styles to perform exercises. Thus, nine volunteer subjects (six men and three women) performed the four mentioned exercises during the same amount of time. The ages of the volunteers ranged between 19 and 32 years. At least four hours before each exercise session, volunteers did not drink substances that alter HR, such as: caffeine, alcohol, nicotine, etc. Five subjects exercise four times a week, while the other four subjects only exercise once in a week. Data was collected between Monday and Friday in the evening (18:00–20:00). Since exercises like push-ups and crunches are generally more physically demanding than jogging and squatting, we designed the sessions of the experiments to consist of one-minute part of exercise and three-minute part of resting. Hence, each volunteer performs at least four different sessions, one per exercise. To avoid unexpected short pauses during the exercising part of each session, hydration needs of the subjects are attended as required. These unexpected pauses would represent noise on the motion signals and can introduce undesired glitches in the training and validation datasets. Such glitches are unavoidable in the practice, but to guarantee the correct labeling of data, we asked subjects to reduce the pauses during exercises. Thus, to overcome this issue, subjects with better physical condition were asked to participate in more than one experiment. By the end of collection, the dataset for training and validation contained over 118,000 three-dimensional samples taken at 20 Hz from the hip-placed accelerometer. 

Consequently, the dataset must be converted to a multidimensional space of features. The considered feature set was the same as in the previous work [26]. Thus, it is shown that the most relevant features are those summarized in Table 3. 

This preselected feature set is the product of an extensive literature review about online HAR systems and the engineering process carried out in [26]. Table 3 describes each considered feature along with their corresponding symbol and meaning. Each one of the 15 mentioned features must be computed from a group of samples, and each array forms a feature vector; therefore, the sample-group size becomes a concern. The group size is named time-window size, since the number of samples required to calculate a feature vector is directly related to the amount of time that the system takes to gather the samples. From the real-time implementation perspective, this time-window size is critical to determine the system latency. Hence, the selected time-window size is one second considering that the perception of activity changes for different users is not immediate and there is the need of gathering enough data within a time window to allow a clear distinction between activities. Thus, the minimum delay for the classifier to detect a change of activities was one second, and each feature vector was computed using 20 samples, due to the sensor 20 Hz sampling frequency.

After setting the time-window size, the dataset was reduced to 5900 feature vectors approximately, each one associated to their respective activity label. Next, classification algorithms to train with this dataset were needed. According to [25], random forest (RF) and k-nearest neighbors (kNN) are the most common choices for online HAR applications. In the present work, both algorithms were used in order to compare their performance to select one for implementation. 

RF algorithm is an estimator that separates the training dataset into subsets for a custom number of decision trees. These trees decide over their respective samples and then the estimator averages their decisions. On the other hand, kNN algorithm maps the feature vectors into a multidimensional space and separates them according to their labels. Then, an incoming sample was compared to its closest training samples (or neighbors), determined by an internal distance measure, and the incoming sample was assigned to the class of most of its neighbors. 

**Figure 3:**
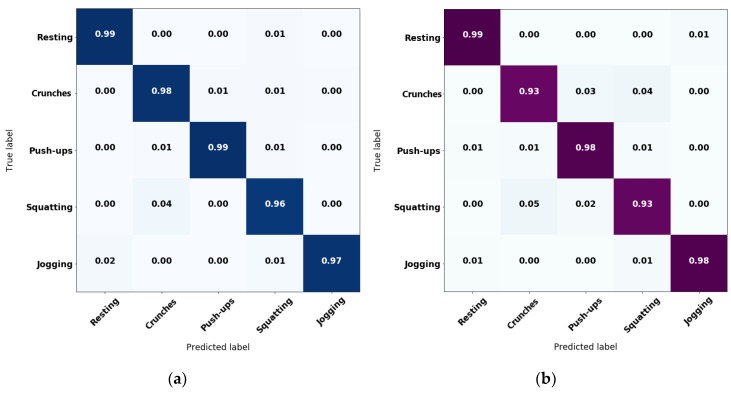
Normalized confusion matrices for: (**a**) random forest (RF) classifier and (**b**) k-nearest neighbor (kNN) classifier.

Collected data from the volunteers was separated by assigning 70% to a training subset and 30% for a validation subset, following a proper data randomization to avoid underfitting. Then, RF estimators were trained varying the number of trees from 2 to 100, and kNN with the number of neighbors from 2 to 50. These values are chosen after noticing that there was not a significant improvement on overall accuracy by increasing the number of trees or neighbors, respectively. Best results show an overall accuracy of 97.7% for RF with 63 trees and 95.2% for kNN with five neighbors. The normalized confusion matrices for both algorithms are displayed in Figure 3. These confusion matrices evince the difference in the overall accuracies by exhibiting less confusion in crunches, push-ups and squatting for the RF algorithm compared to kNN. Such results were obtained using the validation subset. Consequently, optimization efforts were conducted towards RF.

A classifier optimization process is required to reduce dimensionality and, in the case of RF, reduce the number of decision trees. After such a process, validation of the optimized classifier should not show significant reduction on the performance metrics (overall accuracy and confusion matrix).

**Figure 4:**
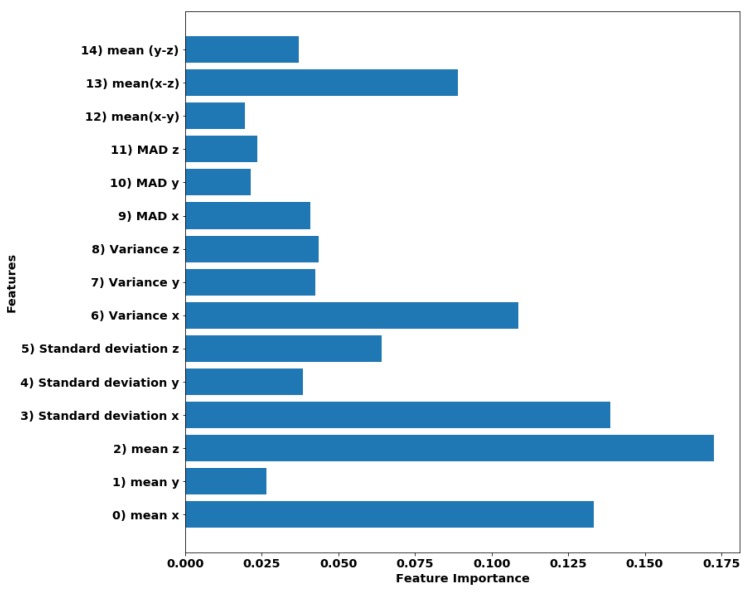
Bar graph of the importance of the features in RF classifier.

For dimensionality reduction, the level of importance that each feature has during training was analyzed. The importance levels considered here were equivalent to Gini importance, which is described in [49]. This importance was computed considering the decrease in average accuracy for the trained trees when a feature value was varied randomly. Thus, significant accuracy detriments point to the significant importance for a feature. In the case of the scikit-learn library, the feature importance levels are normalized. Figure 4 displays a bar graph of the feature importance. As observed, it was clear that $\bar{y}$, MAD(y), MAD(z) and $\bar{x}-\bar{y}$ were the features with the lower significance; and therefore, we proceeded to remove them from the feature vectors. Even though further reduction in the number of features reduced the code size that would be embedded in the mobile application; extra reductions could also compromise the classifier performance. Thus, we decided to work with the new feature set of 11 features; but therefore, the model needs to be retrained with this new set. 

**Figure 5:**
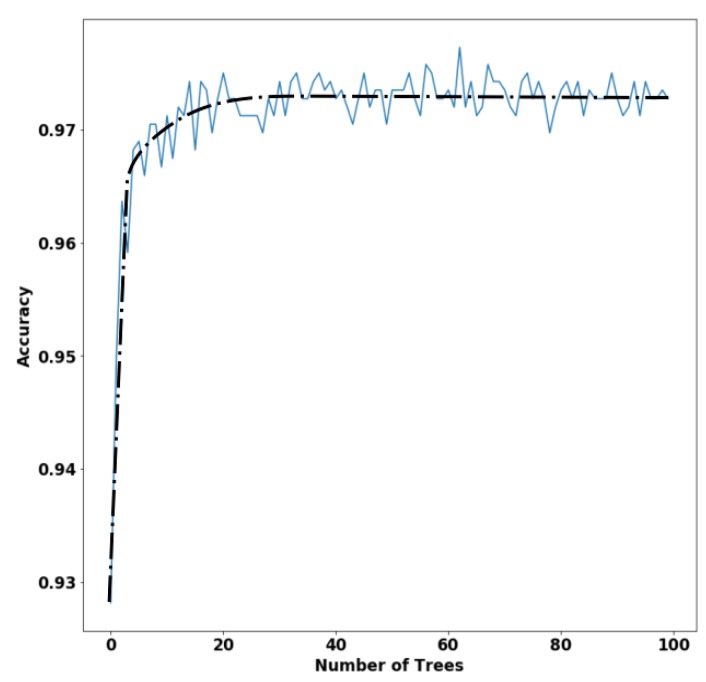
Variation of the overall accuracy with the number of trees in the RF classifier.

**Figure 6:**
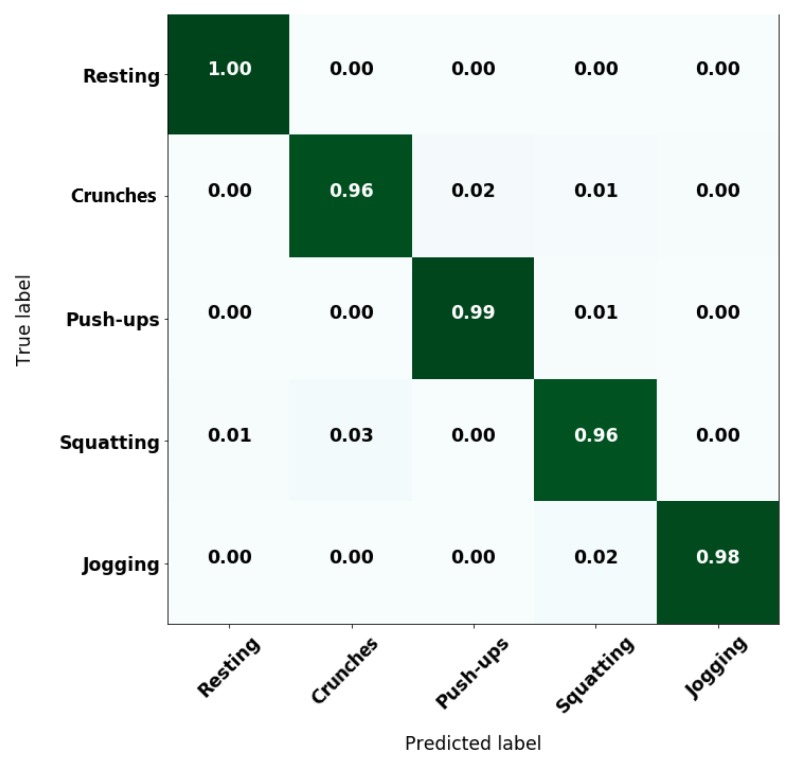
Confusion matrix of the optimized RF classifier.

Figure 5 shows the variation of classifier accuracy with respect to the number of trees, along with a dashed tendency line. According to the accuracy tendency line, after 20 trees, the classifier trended to a stable behavior. Consequently, the number of trees could be reduced to a value above 20 trees without sacrificing performance. In our tests, the overall accuracy with the validation subset changed from 97.7% with 63 trees to 97.5% with 24 trees, but with lower computational cost. Figure 6 exhibits the confusion matrix of this new model, where it could be observed that there was no performance compromise, which facilitated performing the classification directly on the mobile application. 

The resulting model was exported from Python to Java using the Porter tool described in [50] given the requirements of the Android environment. Thus, the model converted into a Java class contains the mathematical description of the 24 decision trees and computes the average decision among them to estimate the corresponding activity. The mobile application includes a testing mode where it reports the true label of the activity performed during the experiment and the labels detected by the model, along with a user identification number and the time stamps of the samples. Thus, this working mode was used for the remaining tests described next.

### 3.2. Online HAR Performance

Once the classifier model was embedded in the mobile application, the model was tested in a real-time environment. For the test, 20 people, different from the nine volunteers who participated in the training data collection, were asked to participate in a new set of experiments. This time, people registered their age on the application along with the average heart rate obtained from the smartwatch on a preliminary 30-seconds resting period. Then, they wore the HAR device on the hip and performed the following exercise routine: push-ups, resting, jogging, resting, squatting, resting, crunches and resting. Each of these activities had a fixed duration of 30 s, which was set seeking a limitation of physical demand to avoid unexpected resting moments. Planned, 30-second resting moments were situated between exercises to help subjects to fulfill the routine without extreme fatigue. Along with each routine, a researcher manipulated the application to set the activity label manually as the subjects shifted from one activity to another. Meanwhile, the system reported to a cloud-stored database the labels obtained from the model, the labels entered manually, a system-custom user identification number and the time stamp.

**Figure 7:**
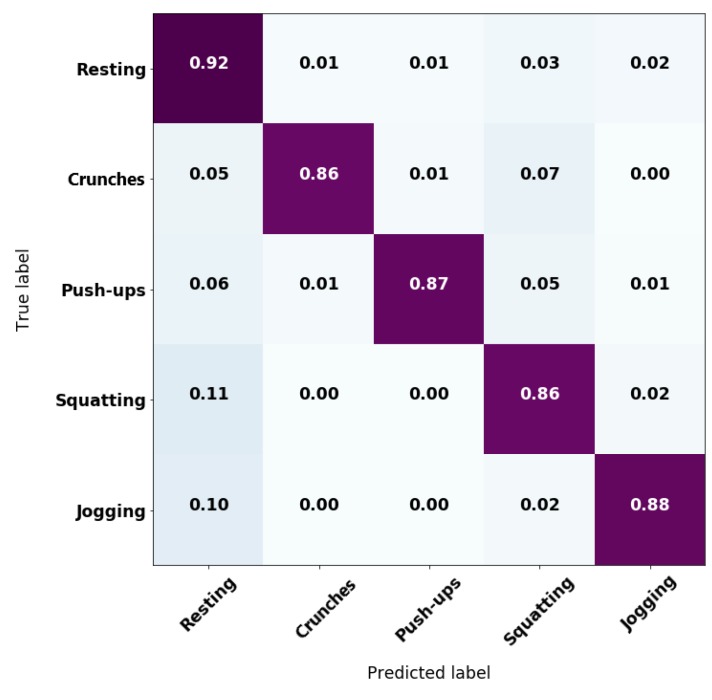
Confusion matrix from testing data.

After this data collection stage, the detected labels were compared against the manual labels to obtain the accuracy rate per activity and per user. Table 4 resumes the statistics of the accuracies from the testing stage. Figure 7 also shows the confusion matrix from online HAR testing. Although the validation accuracy was reported to be 97.5%, real-time tests had an average accuracy between 86% and 92% due to unexpected movements that induced noise for the classifier. Further details regarding the results of Table 4 and Figure 7 are given in the Section Discussion.

### 3.3. Case Study: Physical Workload Evolution on an Individual

**Figure 8:**
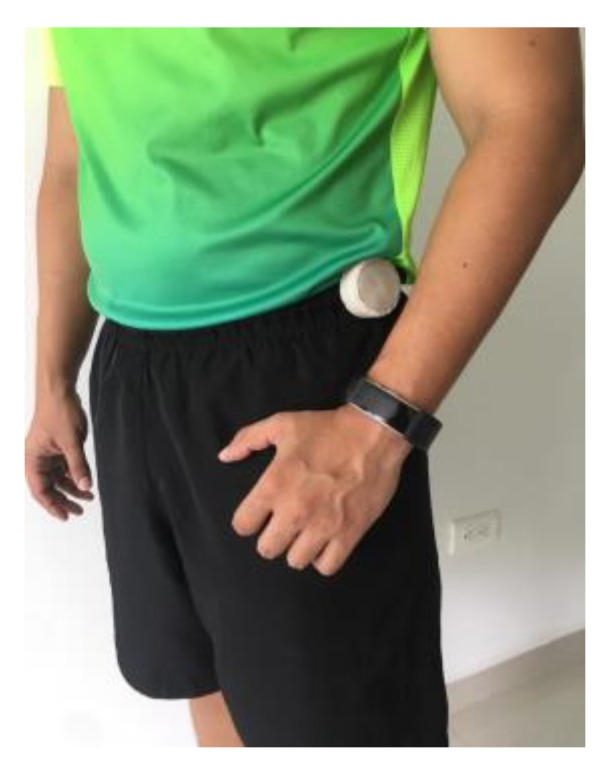
Subject wearing the devices before exercising.

This second case study set its focus on testing the workload estimator reliability. For this purpose, a 27-year-old healthy male subject volunteered to participate in a twenty-day experiment. The subject performed the same exercise routine every day, and its physical workload was recorded. In our system, the collection of workload data was linked to the activity recognition function in order to provide meaningful insights of physical performance. Then, the tests set-up implies that the subject must wear both devices during each session. Figure 8 shows how the subject wears the devices and evinces that they do not represent major discomfort. Before the first session, a preliminary exercising round reveals that crunches do not represent significant physical effort for the subject. Thus, the routine for each day is defined as follows: 15 s of resting to find the reference HR for workload estimation, followed by 60 s of push-ups, 60 s of jogging, 60 s of squatting and 60 s of resting. Then, the one-minute rounds are repeated three times.

The subject did not exercise regularly, which led to the expectation of high levels of workload on the first session and a progressive decline on the physical exigency on successive sessions, as the body adjusts to the exercise routine. The performance of the online HAR was also expected to be steady along the sessions, since the system was used by the same person. Due to the methodology of workload estimation, several Frimat’s scores can be obtained during a one-minute exercise round given the HR variations. However, the system maps those scores into the eight categories, reducing information sensitivity. After each session, a Python script retrieves the classified activities and the true label of activities for HAR assessment, and the workload categories for each activity. Consequently, this script found the statistical mode of the workload categories for an activity and set it as the estimated physical workload.

**Figure 9:**
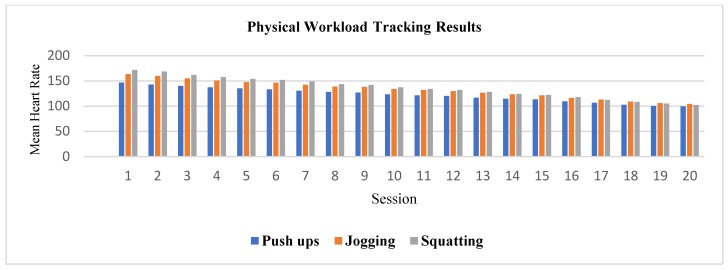
Physical workload tracking results for an individual after 20 days.

**Figure 10:**
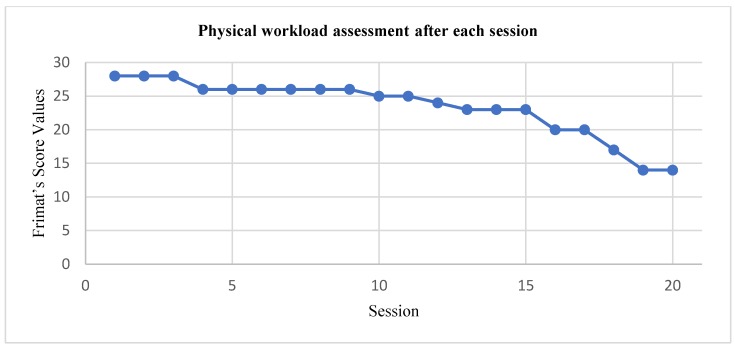
Physical workload assessment after each session.

Figure 9 displays the resulting mean HR for push-ups, squatting and jogging, during each daily session. Figure 10 shows the Frimat’s score values assessment during the resting rounds at the end of each session. These workload values reflect the overall perception of the body of the subject after all the exercising rounds. Complementary, Table 5 resumes the online HAR performance for each session.

## 4. Discussion

This work introduced a system that combines real-time activity monitoring and physical workload estimation to allow remote tracking of workers in physically demanding jobs (as in our previous case study in [19]) and athletes for work health and fitness purposes, respectively. For a comprehensive assessment of system performance, two case studies were presented. The first one embraces the training, validation and real-time testing with 20 subjects of the human activity recognition component. The second case shows the evolution of physical workload for an individual over twenty days.

The training and validation results stood above 95% for overall classification accuracy, compared to previous studies, which also employed wearable devices as shown in Table 6. Critical parameters regarding real-time implementation were considered for comparison such as number of sensors, number of activities and accuracy.

The comparison in Table 6 allows us to locate the present work with an overall accuracy that is only surpassed by a system that only considers three activities and by our previous work in [26]. However, the only work from Table 6 that displays results of real-time tests is [30]. There, tests are 10 s long, compared to the 30 s tests of the present work. Reference [30] considers six test subjects, while our case study considered twenty. Hence, such a length and subject quantity difference can lead to errors in movement data, which makes the results shown in Table 4 generalizable to expected performance during real use.

Additionally, results in Table 4 and Figure 7 containing the average accuracy per activity and their respective standard deviation evinced the tendency of the system to keep a classification exactitude above 85%. However, tests on the first case study were carried by people who do not know the system nor where intensively introduced to its use. Instead, the explanation of the experiment was held short and they were asked to perform the exercises in the most natural way for them. Thus, there were some cases where the subjects took unexpected pauses or just trembled during the exercise, which introduced noise on the one-second time-windows of sampling and reflects on a reduction of the overall accuracy. Nevertheless, by observing at the maximum values, there was also evidence of cases where classification of the embedded model shows no incorrect estimations.

Regarding the second case study, it helps us to validate the reliability of the workload computation in real-time. This approach differs from other workload-related works like [21,22,23,24] where there is no real-time feedback. Instead, authors from [21,22,23,24] take activity and HR data manually and then compute the workload and categorize it. The proposed system does all this process automatically, facilitating the relationship between activity and physical effort, which takes relevance at the application field. The subject considered for the twenty-day experiment of the second case study performed a physically demanding routine that was evaluated as extremely hard at the end of the first ten days, according to Frimat’s criteria. However, a remarkable evolution in the perception of each activity by the subject is shown in Figure 9. In the first sessions, the system evaluated that each type of exercise was extremely hard for the subject, obtaining an average HR of 150 bpm, then in the last session these activities were classified as a light workload, obtaining an average HR of 95 bpm. As expected, the first exercise of the routine (push-ups) displayed the lowest workload amounts, since the body started to adapt to the routine. However, as the exercising round advances, the HR started to increase, which was reflected in higher workloads. Another evidence of the assimilation of the exercising routine was the change from Frimat’s scored values in the resting periods at the end of each session, as shown in Figure 10. Considering that the subject always performed the same exercise routine for twenty sessions (that is, there was no increase or variation in the load), a principle of adaptation is presented in the physical state of the subject [53,54,55]. As can be seen in Figure 9 and Figure 10, at the beginning of the sessions, the physical capacity of the subject was not enough for the established load, but as the exercise sessions increased, the body managed to adapt to that load.

Additionally, Table 5 shows the performance stability of the online HAR component during the second case study. The accuracies stood around 90% for the three activities, considering the fatigue effect on the subject movements. It also must be noticed that these experiments were longer than in the first case study and exhibited higher average accuracy; this is due to the lack of heterogeneity, which leads the system to be exposed to more similar movements each session. Thus, these results confirmed the reliability of the two components of our system for workload tracking purposes.

Finally, the contributions of this work are highlighted as follows:

1. We described the development of a smart physical workload tracking system that allows health and fitness professionals to monitor several people simultaneously and remotely, which is critical in manufacturing and sport industries. To the best of our knowledge, this solution integrating the physical workload concept has not been explored before.
2. We included a well-known concept from ergonomics (Frimat’s criteria) into a real-time e-health application. We achieved this by embedding the workload computation and activity classification on a mobile application, which integrates the signal from a hip-placed accelerometer and a wrist-placed PPG sensor. To the best of our knowledge, this approach has not been presented before.
3. We displayed tests with 20 people performing the same exercise routine. We trained the classification algorithms with data sampled from chosen volunteers; and then tested with a different set of subjects using the devised wearable device during an exercise routine, which comprises crunches, push-ups, squatting and jogging. The accuracy of the classifier was above 85% during real-time testing.
4. We showed the physical progress of a volunteer by tracking his/her physical workload for twenty days while he/she performed the same routine. This case study evidenced that Frimat’s score could provide enough information to determine the level of fitness progress of a person that intends to train using physically demanding exercises.

## 5. Conclusions

A physical workload tracking using human activity recognition and HR measurements with wearable devices was presented. The system used a hip-placed motion sensor and a wrist-placed photoplethysmography sensor for HR. The information from both sensors was gathered by a mobile application through BLE connections; then, performed activity recognition with a trained random forest model and computed physical workload using Frimat’s method. The activity classifier displayed a 97.5% accuracy during validation, and 92% accuracy during real-time tests with 20 subjects. In addition, a twenty-day experiment with a single subject who performed a custom exercise routine shows that the system could recognize the body adaptation to the physically demanding activities.

Future research directions point to a further study of the information relating physical workload and the activities performed. Given the reliability of the wearable-based activity classifier and the workload estimation method, new developments combining ergonomics and machine learning can be carried to predict the amount of physical effort that an activity can represent for a subject. Hence, this could lead to an injury prevention environment powered by historical information on a workplace or physically/mentally demanding tasks.

## Figures and Tables

**Table 1:** Relation between Frimat’s coefficients and cardiac indicators.

Frimat’s Coeffs. Value	Variable Ranges				
	ACC (bpm)	RCC (bpm)	HR*_max_* (bpm)	$\bar{HR}(\mathrm{bpm})$	ΔHR (bpm)
1	10–14	0.10–0.14	110–119	90–94	20–24
2	15–19	0.15–0.19	120–129	95–99	25–29
3	20–24	0.20–0.24	130–139	100–104	30–34
4	25–29	0.25–0.29	140–149	105–109	35–39
5	>30	>0.30	>150	>110	>40

**Table 2:** Ranking of an activity according to its Frimat’s score.

Frimat’s Score Values	Ranking
25	Extremely hard
24	Very hard
22–23	Hard
20–21	Distressing
18–19	Bearable
14–17	Light
12–13	Very light
≤10	Minimum workload

**Table 3:** Features considered for training.

Feature name	Symbol per axis	Meaning
Mean	$\bar{x}$, $\bar{y}$, $\bar{z}$	Statistical tendency of a group of samples from the same axis
Standard deviation	std(x), std(y), std(z)	Measure of variability of a group of samples from the same axis
Variance	var(x), var(y), var(z)	Measure of variability of the squares of a group of samples from their corresponding mean
Mean absolute deviation	MAD(x), MAD(y), MAD(z)	Measure of variability of a group of samples from their corresponding mean
Difference of means	$\overset{=}{x}-\bar{y}$, $\bar{y}-\bar{z}$, $\bar{x}-\bar{z}$	Difference between means of two different axes

**Table 4:** Representative statistics of the online human activity recognition (HAR) testing.

Statistical Parameter	Accuracy Percentages per Activity					
	Resting	Crunches	Push-ups	Squatting	Jogging	Overall accuracy
**Average**	92.26%	86.11%	87.01%	86.71%	87.82%	89.53%
**Standard deviation**	3.34%	7.89%	4.90%	7.53%	6.47%	3.19%
**Maximum**	96.92%	100.00%	96.81%	98.76%	100.00%	95.13%
**Minimum**	84.22%	65.95%	75.24%	70.73%	76.96%	82.69%

**Table 5:** Average accuracies of online HAR for the second case study.

Session	Activity Classification Accuracy		
	Push-ups	Jogging	Squatting
1	94.53%	90.36%	91.38%
2	94.24%	93.83%	89.76%
3	93.03%	91.73%	88.53%
4	92.86%	89.77%	87.76%
5	89.53%	84.20%	90.73%
6	88.50%	87.83%	92.53%
7	88.95%	89.17%	92.67%
8	89.73%	90.13%	91.44%
9	90.64%	90.13%	91.43%
10	91.78%	92.60%	90.14%
11	92.13%	91.27%	90.74%
12	92.16%	90.66%	91.03%
13	91.06%	90.46%	92.80%
14	89.73%	90.36%	90.03%
15	92.63%	90.93%	91.56%
16	92.36%	91.43%	91.03%
17	91.66%	89.43%	92.23%
18	91.23%	90.44%	90.26%
19	90.96%	91.27%	89.43%
20	89.66%	90.13%	93.03%
Average	91.37%	90.31%	90.93%

**Table 6:** Online HAR comparison with previous studies.

Article	Number of Wearable Sensors	Number of Activities	Validation Accuracy
[26]	1	10	98.7%
[27]	5	9	94.8%
[28]	1	5	95.7%
[30]	Smartphone	3	98.6%
[51]	1	9	94.8%
[52]	1	8	95%
This work	1	5	97.5%
